# The Hospital World

**Published:** 1910-08-06

**Authors:** 


### THE HOSPITAL WORLD.

### Personalia.

Mr. Hazlerigg, Mr. Morris' assistant at the London Hospital, has just been appointed secretary to the West London Hospital, Hammersmith, in succession to Mr. Gilbert, who has retired on a pension. The new secretary of the West London is a Cambridge man, who took honours in law and also in classics, and became a solicitor. He then went out to West Africa, where he was for a time engaged under the Crown in legal work, but was forced to return home for health reasons. Since his return he has been actively engaged in institutional work, and has been working at the London Hospital as one of the assistant secretaries and in the Appeal Department. We congratulate Mr. Hazlerigg on his new appointment, which offers excellent scope for his energies and talents as an institutional administrator.

The degree of Master of Laws in the University of Cambridge has been conferred upon Mr. Digby Cotes Preedy, M.A., of Emmanuel College, for a dissertation on "The Legal Relations of Hospital Authorities, their Staff and Patients." Mr. Cotes Preedy was House Physician to St. George's Hospital in 1903, and was for some time Editor of the *Hospital Gazette*.

### Elections and Appointments.

Mr. E. F. Chance has been elected president of the West Bromwich District Hospital. Mr. T. Foley Bache has been appointed hon. secretary, and Alderman Wilson hon. treasurer for the coming year. The Board of Management has been elected as follows: Mr. F. B. Barclay, Mr. C. Beech, Mr. W. T. Davies, Mr. T. Jones, Mr. E. J. Hunt, Mr. J. Moore, Mr. T. H. Spencer, Mr. C. Thomlinson, Mr. S. N. Williams, Mr. S. Woodhall, Colonel G. Walton Walker, and the Rev. M. M. Connor.

Mr. Godfrey Brookes Dixon, M.R.C.S. Eng., L.R.C.P. and L.S.A. Lond., has recently been appointed by the Health Committee of the Birmingham Municipal Authority to the post of medical officer to the Yardley Road Sanatorium for Consumptives. The sanatorium will be opened in the autumn, and is for educational and recuperative purposes. Mr. Dixon held the appointments of house physician to Charing Cross Hospital, London, junior and senior medical officer to the Walton Workhouse and Infirmary, Liverpool, and assistant physician to the Vale of Clwyd Sanatorium, Wales. In addition he has had experience of general practices.

